# Intratumor heterogeneity of driver mutations and TMB distribution in 30 early-stage LUAD patients with multiple lesions

**DOI:** 10.3389/fonc.2022.952572

**Published:** 2022-08-30

**Authors:** Yuan Qiu, Liping Liu, Haihong Yang, Hanzhang Chen, Qiuhua Deng, Dakai Xiao, Yongping Lin, Changbin Zhu, Weiwei Li, Di Shao, Wenxi Jiang, Kui Wu, Jianxing He

**Affiliations:** ^1^ National Clinical Research Center of Respiratory Disease, The First Affiliated Hospital of Guangzhou Medical University, Guangzhou, China; ^2^ State Key Laboratory of Respiratory Diseases, The First Affiliated Hospital of Guangzhou Medical University, Guangzhou, China; ^3^ BGI Genomics, BGI-Shenzhen, Shenzhen, China; ^4^ BGI-Shenzhen, Shenzhen, China

**Keywords:** driver mutation, egfr, tmb, heterogeneity, luad

## Abstract

**Background:**

Differentiating multiple pulmonary lesions as multiple primary lung cancer (MLC) or intra-pulmonary metastasis (IPM) is critical. Lung cancer also has a high genetic heterogeneity, which influenced the treatment strategy. Genetic information may aid in tracing lineage information on multiple lung lesions. This study applied comprehensive genomic profiling to decipher the intrinsic genetics of multiple lung lesions.

**Methods:**

Sixty-six lung adenocarcinomas (LUAD) tumor lesions (FFEP) archived from 30 patients were included in this study. The 508 cancer-related genes were evaluated by targeted next-generation sequencing (MGI-seq 2000).

**Results:**

The study included a total of 30 LUADs (66 samples). The majority of tumors demonstrated intra-tumoral heterogeneity. Two hundred twenty-four mutations were detected by sequencing the 66 samples. We investigated the driver gene mutations of NSCLC patients with multiple lesions. *EGFR* was the most frequently (48/198) mutated driver gene. The codons in *EGFR* mainly affected by mutations were p.L858R (18/66 [27.3%]) and exon 19del (8/66 [12.1%]). In addition, additional driver genes were found, including *TP53, BRAF, ERBB2, MET, and PIK3CA*. We also found that the inter-component heterogeneity of different lesions and more than two different mutation types of EGFR were detected in seven patients with two lesions (P3, P10, P24, P25, P28, P29, and P30). The TMB values of different lesions in each patient were different in 26 patients (except P4, P5, P14, and P30).

**Conclusions:**

Comprehensive genomic profiling should be applied to distinguishing the nature of multiple lung lesions irrespective of radiologic and histologic diagnoses.

## Introduction

The focus of lung cancer treatment shifted significantly with the identification of specific targetable driver mutations. Epidermal growth factor receptor (*EGFR*) driver mutation cancers represent a distinct subset of non-small-cell lung cancer (NSCLC) with broad molecular and clinical heterogeneity (1). For NSCLCs harboring *EGFR* driver mutations, the current standard of treatment in the first-line setting is an *EGFR* tyrosine kinase inhibitor (TKI), either a first- (gefitinib or erlotinib) or second-generation TKI (afatinib). Besides *EGFR, ALK, KRAS, ROS1*, *c-MET*, and *PIK3CA* have been implicated as a driver of NSCLCs, such as crizotinib for *ALK*‐positive lung cancer patients (1, 2). The TP53 gene mutation has also been identified as a driver mutation in NSCLC. These driver mutations are highly heterogeneous, including inter-patient, and intra- and inter-tumor variability. In particular, differentiating multiple pulmonary lesions as multiple primary lung cancer (MLC) or intra-pulmonary metastasis (IPM) is critical (3). Furthermore, a high degree of genetic diversity between the primary lung tumor and corresponding metastatic lesions could play a pivotal role in the therapeutic context of lung cancer patients. Beyond heterogeneity of druggable driver mutations, previous studies have analyzed the presence of mutational signatures across human cancer types, proving that specific mutational signatures correlate with defined tumors (3, 4). The genomics from NSCLC to SCLC have been reported, and molecular characterization of SCLC has revealed an extremely high mutational rate in TP53 and RB1 genes (5).

In contrast, the tumor mutation burden (TMB) reveals the total number of mutations occurring in a tumor specimen and indicates the status of genomic mutations (6). Previous studies have shown that PD-L expression is highly heterogeneous at different locations in the lung. The PD-L1 positive agreement rate between lung primary and metastatic tumors is 63-100%. In 12%-35% of lung cancer patients, PD-L1 expression changes during treatment (7). From driver genes to TMB, what are the genetic characteristics of patients with multiple primary lesions of lung adenocarcinoma?

Multiple somatic alterations lie at the root of cancer development and tumor heterogeneity. This tumor heterogeneity further complicates the design of strategies for effective treatment. Thus, knowledge about the distribution of driver mutations in NSCLCs, particularly in early-stage NSCLCs with multiple lesions, is an area of interest. This study applied comprehensive genomic profiling deciphering intrinsic genetics of multiple lung lesions.

## Methods

### Patients and samples

Between May 2019 and September 2019, 30 patients (many patients with multiple lesions, and only samples meeting the criteria were eventually processed for sequencing) with at least 2 lesions (30) undergoing concurrent surgery were enrolled and sequenced using a targeted exome capture sequencing (568-gene panel) on an MGI-500 platform. Of note, for a patient with multiple lesions, each lesion was evaluated separately, and the outcome of each lesion was reported.

### Comprehensive genomic profiling

Genomic DNA was extracted from FFPE and peripheral blood samples using the Qiagen DNeasy Blood & Tissue Kit (Qiagen, Hilden, Germany) according to the manufacturer’s recommendations. First, raw data generated by the BGISEQ-500 sequencer were filtered by SOAPnuke to exclude reads with low quality. Clean reads were then mapped to the reference human genome (GRCh37/hg19) from a UCSC genome browser. Calling of single nucleotide variants (SNVs) and small insertions/deletions (Ins/Del) was performed with the Genome Analysis Tool kit (GATK) using parameters adapted to HaloPlex-generated sequences. The copy number variants (CNVs) were called using the CNVnator read-depth algorithm. TMB assessed *via* targeted sequencing of **~**1.25Mb broadly recapitulated previous results of the whole exome TMB analysis. Tumor mutation burden was the number of all the non-synonymous mutations/0.7 Mb targeted coding region.

### Statistical analysis

A correlation graph was made using the R Package (version 3.3.0; http://www.r-project.org). A chi‐squared test was used for comparisons of categorical variables across multiple factors. A P< 0.05 was considered significant.

## Results

### Patient characteristics

**Figure 1:**
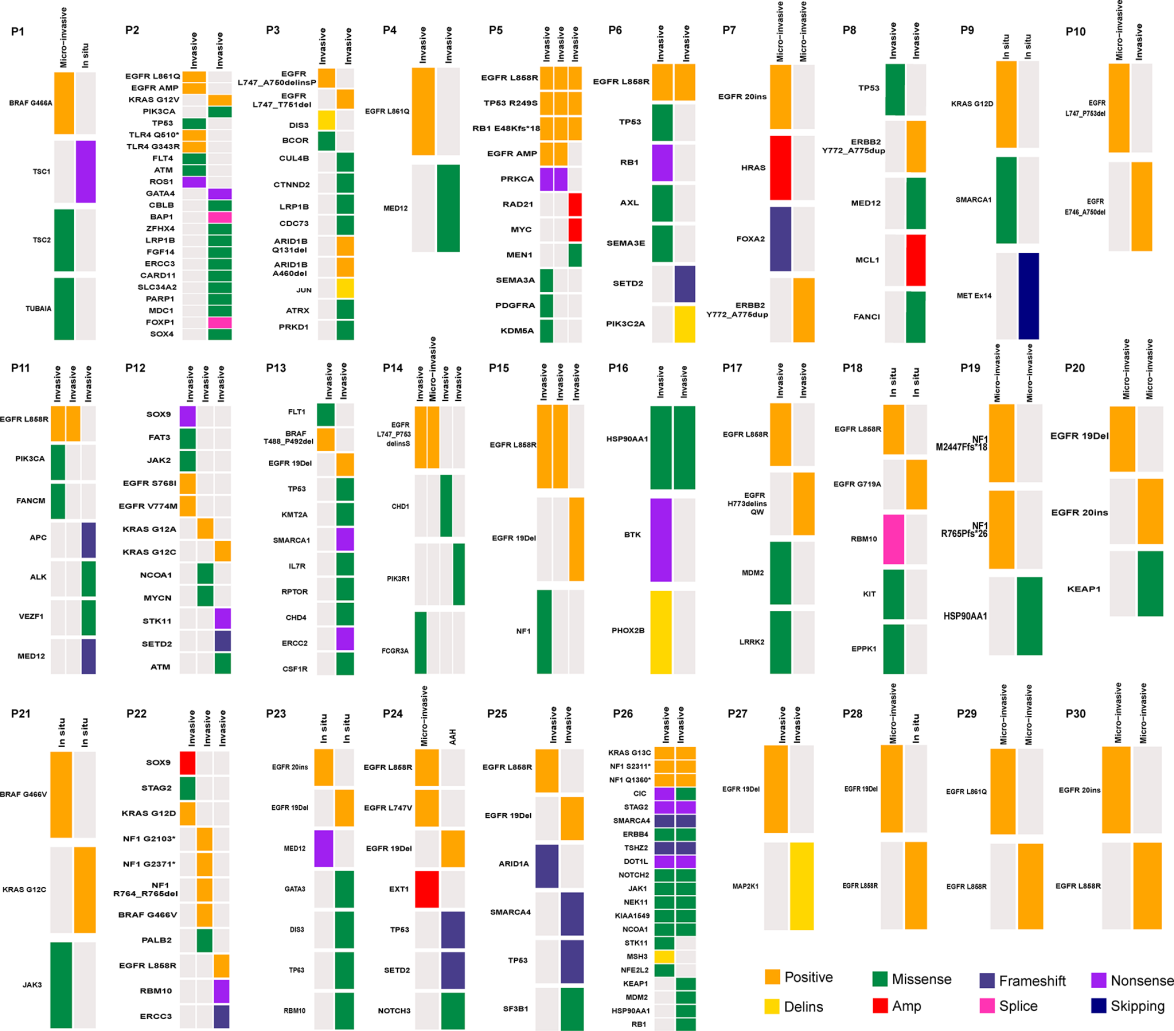
Heatmap showing mutations for each patient.

All 30 LUAD patients had multifocal lesions, which added up to a total of 66 lesions. The clinical features of patients are summarized in **Tables 1**, **2**. The age distribution was between 30 and 69 years. Twenty-two (73.3%) patients were female. Approximately 73.3% (n=22) of the study group were female compared to 26.7% (n=8) male (**Table 1**). Twenty-seven (90.0%) patients had stage IA and three individuals had stage IB and IIB (**Table 1**). Twenty-nine patients (96.7%) had > 2 lung primary lesions, and 1 patient (3.3%) had a metastatic lung lesion. Except for P1, P10, P24, and P28, the pathologic subtypes of all lesions were the same in every patient (**Table 2** and **Figure 1**).

**Table 1:** Clinical characteristics of the patients at baseline.

Characteristic	Overall (N=30)	P value
**Gender**		p>0.05
**Female**	22 (73.3%)	
**Male**	8 (26.7%)	
**Age**		p>0.05
**<60**	14 (46.7%)	
**>=60**	16 (53.3%)	
**Location**		p>0.05
**left**	11 (36.7%)	
**right**	19 (63.3%)	
**Stage**		p<0.05
**IA**	27 (93.3%)	
**IB**	2 (6.7%)	
**Lesion**		p<0.05
**Metastases**	1 (3.3%)	
**Primary**	29 (96.7%)	

**Table 2:** The clinical and driver mutation genes characteristics of 30 patients.

Driver gene	Maximum positive rate (Ⅰ) (N=30)	Positive rate of all lesions (Ⅱ) (N=66)	Minimum positive rate (III) (n=30)
**EGFR**	23 (76.7%)	46 (70.0%)	19 (63.3%)
**ERBB2**	1 (3.3%)	1 (1.5%)	0
**KRAS**	6 (20.0%)	8 (9.1%)	4 (13.3%)
**PIK3CA**	2 (6.7%)	2 (3.0%)	0
**TP53**	7 (23.3%)	8 (12.1%)	6 (20%)
**ALK fusion**	0	0	0
**MET**	1 (3.3%)	1(1.5%)	0

Maximum positive rate, at least one lesion in the patient tested positive; Positive rate of all lesions, positive rate in 66 lesions; Minimum positive rate, all lesions in the patient tested positive.

### Genomic alterations in early-stage NSCLC with multiple lesions

The genomic alterations of each lesion also were reported and the mutations are summarized in **Figure 1**. Altogether, 224 mutations were detected using a 508 gene panel by sequencing the 66 samples. The most common mutations in our study included mutations in *EGFR* (76.7%) and *TP53* (26.7%). Interestingly, we did not find *EML4-ALK* fusion and *MET* mutations. The codons in *EGFR* mainly affected by mutations were p.L858R (18/66 [27.3%]) and exon 19del (8/66 [12.1%]). To explore the genomic origin, we further investigated the shared driver mutations of 30 cases (**Figure 1**).

Four patients (P5, P6, P16, and P26) had shared variations, which occurred in *EGFR* (n=2), *TP53* (n=1), 1 *HSP90AA1* (n=1), and 1 *KRAS* (n=1), and the pathologic outcomes of all lesions were the same in every patient (**Figure 1**). It is worth noting that one of the lesions in P26 was primary, and the other was a metastatic lesion to the lungs (**Figure 1**). In 12 patients (P1, P2, P3, P4, p7, P8, P9, P12, P13, P21, P22, and P27), the pathologic results of the lesion were the same in each patient, but the driver genes were different (**Figure 1**). We also found that the inter-component heterogeneity of different lesions and more than two different mutations type of *EGFR* was detected in seven patients with two lesions (P3, P10, P24, P25, P28, P29, and P30) (**Figure 1**). In P10, P24, and P28, the micro-invasive lesions were found synchronously and the other lesions were subdivided as follows: invasive, Atypical adenomatous hyperplasia (AAH); and *in situ*. Among the 4 lesions in P14 patients, driver mutations (*EGFR* L747_P753delins) were detected in two lesions with different pathologic subtypes, and *CHD1* and *PIK3R1* mutations occurred in two other lesions. In addition, additional driver genes were found, including *ALK, BRAF, ERBB2, MET*, and *PIK3CA.* We also observed that *MED12, TP53*, and *TSC1* gene mutations were detected in patients P4, P8, and P1.

Based on the above results, we analyzed known high-frequency driver mutations and evaluated the maximum and minimum detection rates in different situations (**Table 2**). The results clearly showed that increasing the lesions in one patient increased the mutation rate.

### The distribution of TMB in early-stage NSCLC with multiple lesions

To investigate the intratumor heterogeneity of TMB, we analyzed the TMB status of 30 patients. The clinical features of patients are summarized in **Table 3**.

**Table 3:** The clinical and driver mutation genes characteristics of the two groups.

N-ID	Sample-ID	Stage	Site	Location_1	Location_2	Patho_Subtype	TMB
**P1**	P1-1	IA	primary	left	UL	Micro invasive	2.05
	P1-2	IA	primary	left	LL	In situ	1.54
**P2**	P2-1	IA2	primary	right	UR	invasive	7.69
	P2-2	IA2	primary	right	LR	invasive	5.73
**P3**	P3-1	IA	primary	right	LR	invasive	1.54
	P3-2	IA	primary	right	LR	invasive	4.66
**P4**	P4-1	IA1	primary	left	LL	invasive	2.05
	P4-2	IA1	primary	left	LL	invasive	2.05
**P5**	P5-1	IA	primary	left	UL	invasive	7.69
	P5-2	IA	primary	left	UL	invasive	1.54
	P5-3	IA	primary	left	LL	invasive	7.69
**P6**	P6-1	IA1	primary	right	UR	invasive	3.59
	P6-2	IA1	primary	right	UR	invasive	2.56
**P7**	P7-1	IA1	primary	right	LR	Micro invasive	2.56
	P7-2	IA1	primary	right	LR	Micro invasive	2.05
**P8**	P8-1	IA1	primary	right	UR	invasive	1.54
	P8-2	IA1	primary	right	UR	invasive	0.72
**P9**	P9-1	IA	primary	left	LL	In situ	3.08
	P9-2	IA1	primary	left	UL	In situ	1.03
**P10**	P10-1	IA1	primary	right	LR	Micro invasive	4.62
	P10-2	IA1	primary	right	LR	invasive	1.03
**P11**	P11-1	IA3	primary	right	LR	invasive	3.08
	P11-2	IA3	primary	right	LR	invasive	2.05
	P11-3	IA3	primary	right	LR	invasive	5.64
**P12**	P12-1	IA2	primary	right	LR	invasive	5.13
	P12-2	IA1	primary	right	UR	invasive	5.64
	P12-3	IA3	primary	right	MR	invasive	2.56
**P13**	P13-1	IB	primary	left	UL	invasive	2.56
	P13-2	IA	primary	left	LL	invasive	6.15
**P14**	P14-1	IA	primary	right	LR	Micro invasive	0.51
	P14-2	IA	primary	right	LR	invasive	0.51
	P14-3	IA	primary	right	LR	invasive	1.03
**P15**	P15-1	IA1	primary	right	LR	invasive	2.56
	P15-2	IA1	primary	right	UR	invasive	0.51
	P15-3	IA1	primary	right	LR	invasive	2.05
**P16**	P16-1	IA3	primary	right	UR	invasive	1.03
	P16-2	IA1	primary	right	UR	invasive	1.79
**P17**	P71-1	IAb	primary	left	UL	invasive	1.54
	P17-2	IAb	primary	left	UL	invasive	2.56
**P18**	P18-1	IA	primary	left	UL	invasive	1.54
	P18-2	IA	primary	left	UL	invasive	1.43
**P19**	P19-1	IA1	primary	right	UR	Micro invasive	1.03
	P19-2	IA1	primary	right	UR	Micro invasive	1.54
**P20**	P20-1	IA1	primary	right	LR	Micro invasive	2.05
	P20-2	IA1	primary	right	LR	Micro invasive	0.51
**P21**	P21-1	IA1	primary	left	UL	In situ	8.72
	P21-2	IA1	primary	left	UL	In situ	1.03
**P22**	P22-1	IB	primary	right	UR	invasive	3.59
	P22-2	IB	primary	right	UR	invasive	2.05
	P22-3	IA3	primary	left	UL	invasive	1.08
**P23**	P23-1	IA1	primary	right	LR	In situ	0
	P23-2	IA1	primary	right	LR	In situ	1.35
**P24**	P24-1	IA2	primary	right	UR	Micro invasive	2.05
	P24-2	IA1	primary	right	UR	/	5.64
**P25**	P25-1	IA2	primary	right	MR	invasive	3.08
	P25-2	IA2	primary	right	UR	invasive	2.05
**P26**	P26-1	IA1	metastases	right	UR	invasive	7.69
	P26-2	IA1	metastases	right	LR	invasive	8.72
**P27**	P27-1	IA1	primary	right	UR	invasive	4.1
	P27-2	IA1	primary	left	LL	invasive	2.05
**P28**	P28-1	IA1	primary	left	UL	Micro invasive	2.56
	P28-2	IA1	primary	left	LL	In situ	1.54
**P29**	P29-1	IA1	primary	left	LL	Micro invasive	2.05
	P29-2	IA1	primary	right	UR	Micro invasive	2.56
**P30**	P30-1	IA1	primary	right	UR	Micro invasive	0.51
	P30-2	IA1	primary	right	UR	Micro invasive	0.51

UL, uplift; UR, upright.

We found that the TMB values of different lesions in each patient were different in 26 (except P4, P5, P14, and P30; **Table 3**). Among the 26 patients, 4 (P1, P10, P24, and P28; **Table 3**) with 2 lesions, and all lesions in each patient had a different pathologic subtype. It is worth noting that the sampling locations of each lesion in P1 and P28 were also different (**Table 3**). In P5 and P14 with 3 lesions, the distribution of TMB is also more diverse. Two lesions in N5 patients (P5-1 and P5-3) had the same TMB value, stage, and pathologic classification. In the remaining lesion (P5-2), a different TMB value and sampling location were observed (**Table 3**). A similar phenomenon had also been found in P14. The lesions with different pathologies (P14-1 and P14-2) had the same TMB value, and the lesions with different TMB values (P14-1 and P14-3) had different TMB values. The clinical features and TMB values of all lesions in each patient (P4 and P30) were the same. Among two metastatic lesions from one patient (P1), highly heterogeneous TMB values were found in two lesions with the same driver gene height (**Figure 1** and **Table 3**).

## Discussion

Tumor heterogeneity is frequently cited as a confounding factor and limitation in molecular studies of tumors (8). The intra-tumor heterogeneity of multiple primary lung cancers (MPLC) may lead to therapy failure and cancer progression (9, 10). Further study of the molecular characteristics and TMB distribution of MPLC can help guide clinical practice effectively.

Previous studies have demonstrated that approximately 85% of individuals with LUAD have known driver mutations, including *EGFR, KRAS, ALK, ERBB2, ROS1, RET, MET, BRAF, NRAS*, and *TP53* (11, 12). Our study showed that the main common driver gene was EGFR. The available data also showed that early‐ and advanced‐stage LUAD exhibit the same *EGFR* mutation frequencies and types (13). The major driver mutations other than *EGFR, KRAS, ERBB2, PIK3CA*, and *TP53* were not detected in our study, which more frequently occurs in Asian LUAD (12, 14). This may be because *ALK* fusion gene-positive LUAD typically occurs in young non-smokers or rare smokers (15). METex14 alterations are enriched in sarcomatoid histologies, with a prevalence ranging from 8%-22% (16, 17). The *ROS1* gene was reported to be present in 1%–2% of NSCLCs, which is common in young non-smoking female patients with LUAD (17). These findings indicate that known driver gene mutations dominate early genetic events and conferred a selective growth advantage. These mutations occurred in multiple primary and metastatic lesions of the same patient. Interestingly, if only one lesion was detected, the lowest detection rate may occur. Therefore, the simultaneous detection of multiple lesions helps to increase the probability of targeting drugs. Our key finding is that the different *EGFR* mutations always occur in different lesions of one patient, suggesting that heterogeneous distribution of *EGFR* mutation is the major driver event in the development of LUAD. The intertumoral heterogeneity of *EGFR*-activating mutations has also been confirmed at the single-cell level, which was associated with the *EGFR*-TKI response in LUAD patients harboring the *EGFR* L858R mutation (9). The resistance led by the T790M mutation is more attributed to 19 exon del than L858R (18). When the patients have a double mutation with exon 19del and T790M or exon 19del and L858R, patients will not benefit from first-generation *EGFR*-targeted TKI drugs and may benefit from AZD9291 (19). Identical tumor subtypes respond differently to the same drug, which may be among many others an effect of intratumoral heterogeneity. We also identified *HSP90AA1* mutations in one patient with two lesions. It has been reported that *HSP90AA1* is associated with a shorter overall survival rate in patients with NSCLC (20). Molecular heterogeneity between multiple lesions in individuals with the same pathologic type represents different biological processes, resulting frequently in different treatment responses for each patient. So, we further speculated whether multi-primary tumors enrich tumor heterogeneity, and thus have a greater impact on the distribution of TMB.

TMB sketches out the status of genomic mutations (21), which is emerging as a practical biomarker for predicting the response of immune checkpoint inhibitors (ICIs) (22). Early studies have shown that PD-L1 expression was markedly different between primary tumors and paired metastatic lymph nodes (23). Different histologic components within a tumor and different pathologic features contribute to the heterogeneous PD-L1 expression in patients with NSCLC (24). By exploring the TMB distribution in different lesions of the intratumor, our results showed that in one patient, TMB distribution in different lesions was diverse. Although the pathologic phenotypes of the multiple lesions were consistent, the TMB distribution was still quite different. Thus, heterogeneity within a tumor and across multiple tumors within a patient was further demonstrated. Currently, most clinical trials do not account for intratumoral heterogeneity of TMB. Intratumor heterogeneity may contribute to the ambiguous clinical results on TMB status as a predictor for immunotherapy response in patients with LUAD.

Morphologic heterogeneity of the tumor may sometimes provide an important clue to genomic heterogeneity of the tumor, which is often associated with responses to particular molecular-targeted and immunotherapy therapy. It also likely reflects the fact that, because of the considerable tumor, patient, and treatment heterogeneity, no one “optimal” management strategy can be delineated. This shows that the simultaneous detection of multiple primary or metastasis lesions that meet the sequencing standards can help provide more clinical guidance information.

## Data availability statement

The datasets presented in this study can be found in online repositories. The names of the repository/repositories and accession number(s) can be found below: https://db.cngb.org/, CNP0001479.

## Ethics statement

The studies involving human participants were reviewed and approved by Institutional Review Board (IRB) of the First Affiliated Hospital of Guangzhou Medical University. The patients/participants provided their written informed consent to participate in this study.

## Author contributions

JH, YQ, and KW conceived the study. LL, HY, HC, QD, DX, YL, and WJ collected study materials or patients. DS, WL, and CZ performed data cleaning and statistical analysis. WL, CZ, and DS wrote the manuscript. All authors read and approved the final manuscript.

## Funding

This study is funded by the Foundation and Applied Basic Research Fund of Guangdong Province (2020A1515011293) and the National Natural Science Foundation of China (Grant No. 81772486).

## Conflict of interest

CZ, WL, DS, and WJ are employees of BGI Genomics which produces the panel test used in this study.

The remaining authors declare that the research was conducted in the absence of any commercial or financial relationships that could be construed as a potential conflict of interest.

## Publisher’s note

All claims expressed in this article are solely those of the authors and do not necessarily represent those of their affiliated organizations, or those of the publisher, the editors and the reviewers. Any product that may be evaluated in this article, or claim that may be made by its manufacturer, is not guaranteed or endorsed by the publisher.
